# Non-alcoholic Fatty Liver Disease and the Risk of Incident Atrial Fibrillation in Young Adults: A Nationwide Population-Based Cohort Study

**DOI:** 10.3389/fcvm.2022.832023

**Published:** 2022-03-23

**Authors:** JungMin Choi, So-Ryoung Lee, Eue-Keun Choi, Hyo-Jeong Ahn, Soonil Kwon, Sang-Hyeon Park, HuiJin Lee, Jaewook Chung, MinJu Han, Seung-Woo Lee, Kyung-Do Han, Seil Oh, Gregory Y. H. Lip

**Affiliations:** ^1^Department of Internal Medicine, Seoul National University Hospital, Seoul, South Korea; ^2^Department of Internal Medicine, Seoul National University College of Medicine, Seoul, South Korea; ^3^Department of Medical Statistics, College of Medicine, Catholic University of Korea, Seoul, South Korea; ^4^Department of Statistics and Actuarial Science, Soongsil University, Seoul, South Korea; ^5^Liverpool Center for Cardiovascular Science, University of Liverpool and Liverpool Chest and Heart Hospital, Liverpool, United Kingdom; ^6^Department of Clinical Medicine, Aalborg University, Aalborg, Denmark

**Keywords:** non-alcoholic fatty liver disease, fatty liver index, atrial fibrillation, young, adult

## Abstract

**Background:**

Non-alcoholic fatty liver disease (NAFLD) is a multisystem disease including cardiovascular. However, the association between NAFLD and the risk of incident atrial fibrillation (AF), especially in young adults, remains unclear. We aimed to evaluate the association between NAFLD as assessed by the fatty liver index (FLI) and the risk of AF in young adults.

**Methods:**

We identified individuals aged 20–39 years who underwent health examinations conducted by the Korean National Health Insurance Corporation between January 2009 and December 2012. Individuals with significant liver disease, heavy alcohol consumption, or prevalent AF were excluded. We categorized based on FLI: <30, 30 to <60, and ≥60. Incident AF was evaluated as the primary outcome.

**Results:**

We included 5,333,907 subjects (mean age, 31 ± 5 years; men, 57%). During a mean follow-up of 7.4 ± 1.1 years, 12,096 patients had newly diagnosed AF (incidence rate 0.31 per 1,000 person-years). After adjustment, subjects with FLI 30 to <60 and FLI ≥60 showed a higher risk of AF compared to those with FLI <30 (hazard ratio [HR] 1.21, 95% confidence interval [CI, 1.15–1.27] and HR 1.47, 95% CI [1.39–1.55], *p* < 0.001, respectively). In women, the increased AF risk was accentuated in the higher FLI group than in the individuals with FLI <30, compared with men (*p*-for-interaction = 0.023). A higher incident AF risk in the higher FLI groups was consistently observed in various subgroups.

**Conclusion:**

Among young adults, NAFLD assessed using FLI was positively correlated with the AF risk. These findings support the evidence of AF screening in young adults with high FLI scores.

## Introduction

Atrial fibrillation (AF) is the most common arrhythmia, with a healthcare burden that is expected to continuously increase worldwide (1, 2). AF increases the risk of all-cause mortality and morbidity than in the general population, especially in young patients (3, 4). It is a significant risk factor for acute ischemic stroke, although it is relatively uncommon in young adults (5).

Even though some risk factors differentiate among different age groups, Non-genetic risk factors such as obesity, smoking, and hypertension are consistently associated with the risk of incident AF irrespective of age group (6). Consequently, recent studies have focused on evaluating risk factors of AF as a cluster of metabolic disease (7–10). Non-alcoholic fatty liver disease (NAFLD) is one of the factors being evaluated as a possible surrogate marker for such metabolic unhealthiness.

NAFLD is a disease of epidemic proportions in high-income countries being responsible for approximately one-quarter of the global population, up to 30% in Korea (11). The prevalence and burden of NAFLD are expected to rise, especially in young adults (12). The associations between ultrasonography diagnosed NAFLD and an increased risk of AF have been reported (13–15). However, the association between NAFLD as assessed by the fatty liver index (FLI) with risk of AF in young adults in a large population remains unknown. FLI is a surrogate marker with validated accuracy of 0.84 (95% confidence interval [CI] 0.81–0.87) in detecting fatty liver, (16) and is used for screening NAFLD in epidemiological studies of the general population (17–19).

Therefore, this study aimed to evaluate the association between NAFLD assessed by the FLI and the risk of AF in young adults aged 20–39 years in a large nationwide population-based cohort study.


$$\begin{array}{l} \text{FLI}=\frac{\text{e}0.953\times \text{loge}\left(\text{TG}\right)+0.139\times \text{BMI}+0.718\times \text{loge}\left(\text{GGT}\right)+0.053\times \text{waist circumference}-15.745}{1\;+\text{ e}0.953\times \text{loge}\left(\text{TG}\right)+0.139\times \text{BMI}+0.718\times \text{loge}\left(\text{GGT}\right)+0.053\times \text{waist circumference}-15.745} \end{array}$$

## Methods

This study used all the data from claims database, the Korean National Health Insurance Service (NHIS). The NHIS in South Korea covers over 50 million people residing in South Korea. Among the total population, the biannually employed, household and 20-year-old or older dependent family members (biannually), and medical aid beneficiary householders aged 19–64 years (biannually) are the candidates of the general National Health Screening Program of Korea. The National Health Screening Program of Korea consists of seven categories, including physical examination and other laboratory examinations (blood tests, urine examination, and chest radiography), and ~74.1% of the candidates participated in 2019 (20). The database by the National Health Information Database contains information on not only demographic variables and date of death, but also information on health behaviors (21, 22). Our study used data collected between January 1, 2009, and December 31, 2012.

This study was exempted from institutional review board (IRB) review (IRB no. E-2105-116-1219) as the study used anonymized data. The informed consent could not be acquired as the database was de-identified when provided from the NHIS. The approval for the use of the released database from 2009 to 2012 by the NHIS was received in 2021.

### Study Population

**Figure 1:**
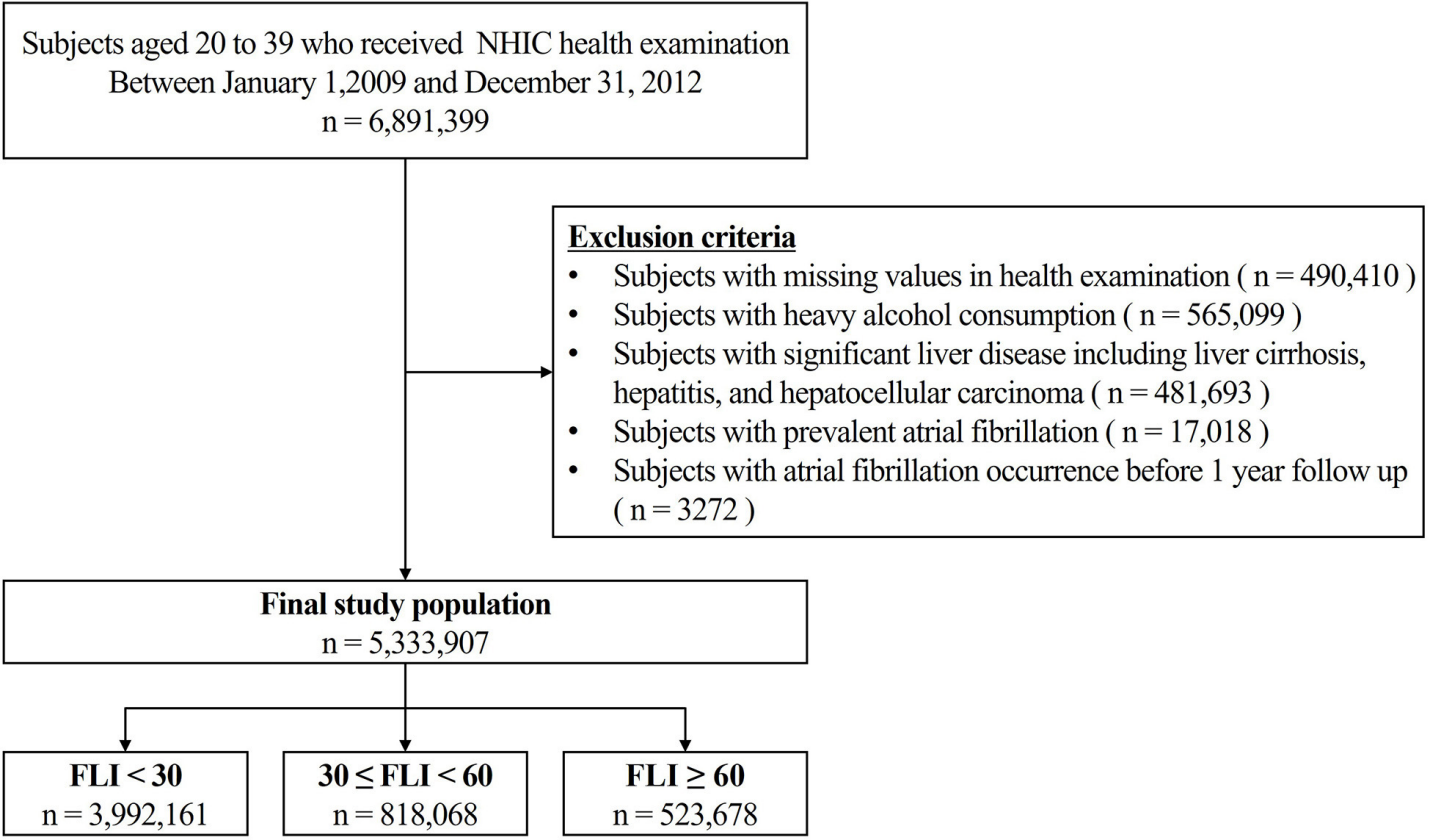
Overview of the Patient flow. NIHC, National health insurance corporation; FLI, fatty liver index.

Subjects aged 20–39 years who underwent health examinations conducted by the Korean National Health Insurance Corporation between January 1, 2009, and December 31, 2012, were identified (*n* = 6,891,399). We excluded patients with heavy alcohol consumption (>30 g/day, *n* = 565,099; 8.2%) based on the self-reported questionnaire in a health examination, significant liver disease (liver cirrhosis, hepatitis, and hepatocellular carcinoma) and those with prevalent AF before enrollment (Figure 1).

### Definition of Non-alcoholic Fatty Liver Disease

In this study, FLI was used to define NAFLD. The standard diagnosis of NAFLD requires hepatic steatosis confirmation by histologic or radiographic examination in the absence of excessive alcohol consumption and other liver diseases (23, 24). The gold standard for diagnosing NAFLD is liver biopsy, but ultrasonography is also reported to have reliability and accuracy in diagnosing NAFLD with area under the summary receiving operating characteristics curve of 0.93 (25). However, liver biopsy and ultrasonography have limited usability in screening for NAFLD, especially in a large population study.

In this context, the FLI proposed by Bedogni et al. is an alternative option for diagnosing NAFLD as the formula contains body mass index (BMI), waist circumference, triglyceride (TG), and gamma glutamyl transpeptidase (GGT), which are easy to obtain (16).

In a previous study which validated FLI values using fatty liver diagnosed by ultrasonography, a an FLI <30 (negative likelihood ratio = 0.2) rules out and a an FLI ≥60 (positive likelihood ratio = 4.3) rules in fatty liver (16). This cut-off value has been used widely in other populations (18, 19, 26). Following these cut-offs, in our study, we set the group with FLI <30 who were less likely to have a clinical fatty liver as a reference group, and those with FLI ≥60 were defined as patients with NAFLD. Considering that FLI reflects hepatic steatosis and insulin resistance, we assumed that FLI has a meaning of the metabolic healthiness of individuals as a continuous variable, even though FLI value of 30 to <60 is under the NAFLD cutoff. In previous studies, not only the patients with NAFLD (FLI ≥60), but those with FLI 30 to <60 also had a higher risk of diabetes mellitus or cardiovascular diseases compared to those with FLI <30 (27, 28). Therefore, we analyzed patients with FLI 30 to <60 as an independent group. In consideration of FLI being a continuous variable, we also used the decile groups of the study population for a supplementary analysis: FLI < 1.94, FLI < 3.04, FLI < 4.53, FLI < 6.74, FLI < 10.16, FLI < 15.56, FLI < 24.16, FLI < 37.82, FLI < 59.51, 59.51 ≤ FLI.

### Covariates

Comorbidities were defined using the International Classification of Disease, Tenth, Clinical Modification (ICD-10-CM) codes. Hypertension and diabetes mellitus were defined with an additive requirement of a minimum of one prescription of anti-hypertensive or anti-diabetic drugs, respectively (22). Detailed information on the definition of each covariate is summarized in Supplementary Table 1. The general health examination included systolic and diastolic blood pressure, weight, height, BMI, and waist circumference. The laboratory results consisted of fasting glucose, estimated glomerular filtration rate (eGFR), total cholesterol, TG, high-density lipoprotein cholesterol (HDL-C), and low-density lipoprotein cholesterol (LDL-C) (22, 29, 30). The self-reported questionnaire consisted of information on smoking behavior (never, ex-, or current), alcohol consumption behavior (none, mild to moderate, or heavy), and regular exercise (22, 31, 32). The low income was defined as the lowest 20% among the entire Korean population and those supported by medical aid.

### Study Outcomes

Incident AF was defined as the first occurrence of AF during at least two different days of hospital visits (outpatient) or the first admission or death with ICD-10-CM codes (I48; AF and atrial flutter). The use of I48 as a definition of AF have been validated with a positive predictive value of 94.1% (33). The index year was defined as the first NHIS health examination, and those who developed AF before 1 year from the index year were considered irrelevant to NAFLD and were excluded. Data were censored at the time of the event, whichever came first: incidence of AF, disqualification from the NHIS (immigration or death), or end of the study (December 31, 2018).

### Statistical Analysis

The descriptive values of the baseline characteristics were described as percentages for categorical data and mean ± standard deviation for continuous variables. The serum TG level was presented as a geometric mean (95% CI). One-way analysis of variance and chi-square tests were performed to evaluate the differences between the FLI groups. *Post hoc* tests for pairwise comparisons were performed.

The incidence rate (IR) of AF was measured as the number of incident AF events per 1,000 person-years. Kaplan-Meier analyses were used to plot the cumulative incidences of AF in the FLI groups, with a log-rank *p*-value for the comparison of statistical significance between the groups. Cox proportional hazards regression analysis was used to estimate hazard ratios (HRs) and 95% CIs of AF incidence. A total of three models with different combinations of covariates were used for the evaluation: unadjusted model (model 1), model adjusted for age and sex (model 2), model adjusted for age, sex, smoking, alcohol consumption, regular exercise, diabetes mellitus, hypertension, dyslipidemia, heart failure, prior ischemic stroke, prior myocardial infarction, chronic obstructive pulmonary disease, chronic kidney disease, sleep apnea, hyperthyroidism, and low income (Model 3).

Subgroup analyses were performed according to age (<30 and ≥30 years), sex, obesity (BMI <25 and BMI ≥25), central obesity (waist circumference: men <90 cm, women <85 cm, and men ≥90 cm or women ≥85 cm), alcohol consumption (none and mild), and income (low and others).

We performed sensitivity analyses to provide complementary results for the healthy population who did not have significant cardiovascular comorbidities, such as hypertension, diabetes mellitus, dyslipidemia, heart failure, and prior ischemic stroke and myocardial infarction at baseline.

Statistical significance was set at *p* < 0.05. All statistical analyses were performed using SAS version 9.4 (SAS Institute, Cary, North Carolina).

## Results

A total of 5,333,907 patients were finally included in this study and categorized into three groups: FLI <30 (*n* = 3,992,161, 75%), FLI 30 to <60 (*n* = 818,068, 15%), and FLI ≥ 60 (*n* = 523,678, 10%). The subjects in the higher FLI group were men and showed a higher prevalence of comorbidities, including hypertension, diabetes mellitus, dyslipidemia, heart failure, prior ischemic stroke, previous myocardial infarction, and sleep apnea. The higher FLI groups had a higher proportion of current smokers and subjects with mild alcohol consumption and higher mean BMI and waist circumference levels than the lower FLI groups. In addition, patients with higher FLI values showed higher fasting glucose, total cholesterol, LDL-C, TG, and lower HDL-C than those with lower FLI values. Low income was more common in the lower FLI group (Table 1). For pairwise comparisons, Tukey's *post hoc* test revealed a significant difference (*P* < 0.001) between each pair of groups in all variables.

**Table 1:** Baseline characteristics of the study population.

	**Total** **(*n* = 5,333,907)**	**FLI**			***P*** **-value**
		** <30** **(*n* = 3,992,161)**	**30 to <60** **(*n* = 818,068)**	**≥60** **(*n* = 523,678)**	
Age, years					
Mean ± SD	30.7 ± 5.0	30.1 ± 5.1	32.5 ± 4.4	32.8 ± 4.3	<0.001
<30	43.1	48.8	27.8	23.9	<0.001
≥30	56.9	51.2	72.2	76.1	
Sex (men)	56.5	45.2	88.7	92.3	<0.001
Comorbidities					
Hypertension	6.6	3.2	11.6	24.0	<0.001
Diabetes mellitus	1.7	0.9	2.7	6.9	<0.001
Dyslipidaemia	6.4	3.2	11.8	22.8	<0.001
Heart failure	0.1	0.0	0.1	0.1	<0.001
Prior ischemic stroke	0.1	0.0	0.1	0.1	<0.001
Prior MI	0.1	0.1	0.1	0.1	<0.001
CKD	2.7	2.7	2.8	2.6	<0.001
COPD	2.5	2.6	2.4	2.5	<0.001
Sleep apnea	0.07	0.05	0.13	0.21	<0.001
Thyroid disease	1.6	1.8	1.1	0.9	<0.001
Social history					
Smoking					<0.001
Non-smoker	58.5	66.9	36.2	28.8	
Ex-smoker	9.7	8.2	14.7	13.8	
Current smoker	31.8	24.9	49.1	57.4	
Alcohol consumption					<0.001
Non-drinker	41.2	45.1	30.7	27.4	
Mild (0–30 g/day)	58.8	54.9	69.3	72.6	
Regular exercise	12.5	12.2	13.8	13.0	<0.001
Low income	16.4	17.8	12.5	12.0	<0.001
Health examination					
SBP (mmHg)	117.1 ± 13.0	114.5 ± 12.0	123.1 ± 12.0	128.2 ± 13.4	<0.001
DBP (mmHg)	73.4 ± 9.3	71.6 ± 8.6	77.2 ± 8.8	81.0 ± 9.9	<0.001
BMI (kg/m^2^)					
Mean ± SD	22.8 ± 3.6	21.5 ± 2.5	25.7 ± 2.3	28.9 ± 3.4	<0.001
≥25	24.8	8.6	61	91.3	<0.001
WC (cm)					
Mean ± SD	76.9 ± 10.2	73.1 ± 7.4	85.5 ± 5.4	93.1 ± 9.9	<0.001
Men ≥ 90, Women ≥ 85	12.0	2	25.2	67.7	<0.001
Laboratory results					
eGFR (mL/min/1.73 m2)	96.1 ± 49.8	96.9 ± 48.3	93.8 ± 53.6	93.7 ± 54.5	<0.001
Fasting Glucose (mg/dL)	90.5 ± 16.0	88.7 ± 12.6	93.7 ±18.6	99.4 ± 27.3	<0.001
Total cholesterol (mg/dL)	183.9 ± 35.9	177.3 ± 32.4	198.5 ± 36.5	211.6 ± 39.8	<0.001
HDL-C (mg/dL)	57.7 ± 25.6	60.0 ± 22.3	51.9 ± 32.2	49.2 ± 33.2	<0.001
LDL-C (mg/dL)	104.8 ± 34.5	101.0 ± 31.2	115.9 ± 38.7	115.9 ± 43.4	<0.001
^*^TG (mg/dL)	93.9 (93.9–94.0)	76.2 (76.1–76.2)	150.8 (150.6–150.9)	221.8 (221.5–222.1)	<0.001

*Categorical variables were presented as a percentage and continuous variables were presented as mean and standard deviation. For pairwise comparisons, Tukey's post hoc test revealed a significant difference (P < 0.001) between each pair of groups in all variables*.

* *TG was presented as geometric mean (95% confidence interval)*.

*BMI, body mass index; CKD, chronic kidney disease; COPD, chronic obstructive pulmonary disease; DBP, diastolic blood pressure; eGFR, estimated glomerular filtration rate; FLI, fatty liver index; HDL-C, high density lipoprotein-cholesterol; LDL-C, low density lipoprotein-cholesterol; MI, myocardial infarction; SBP, systolic blood pressure; TG, triglyceride; WC, waist circumference*.

### Risk of Incident AF According to FLI Values

**Figure 2:**
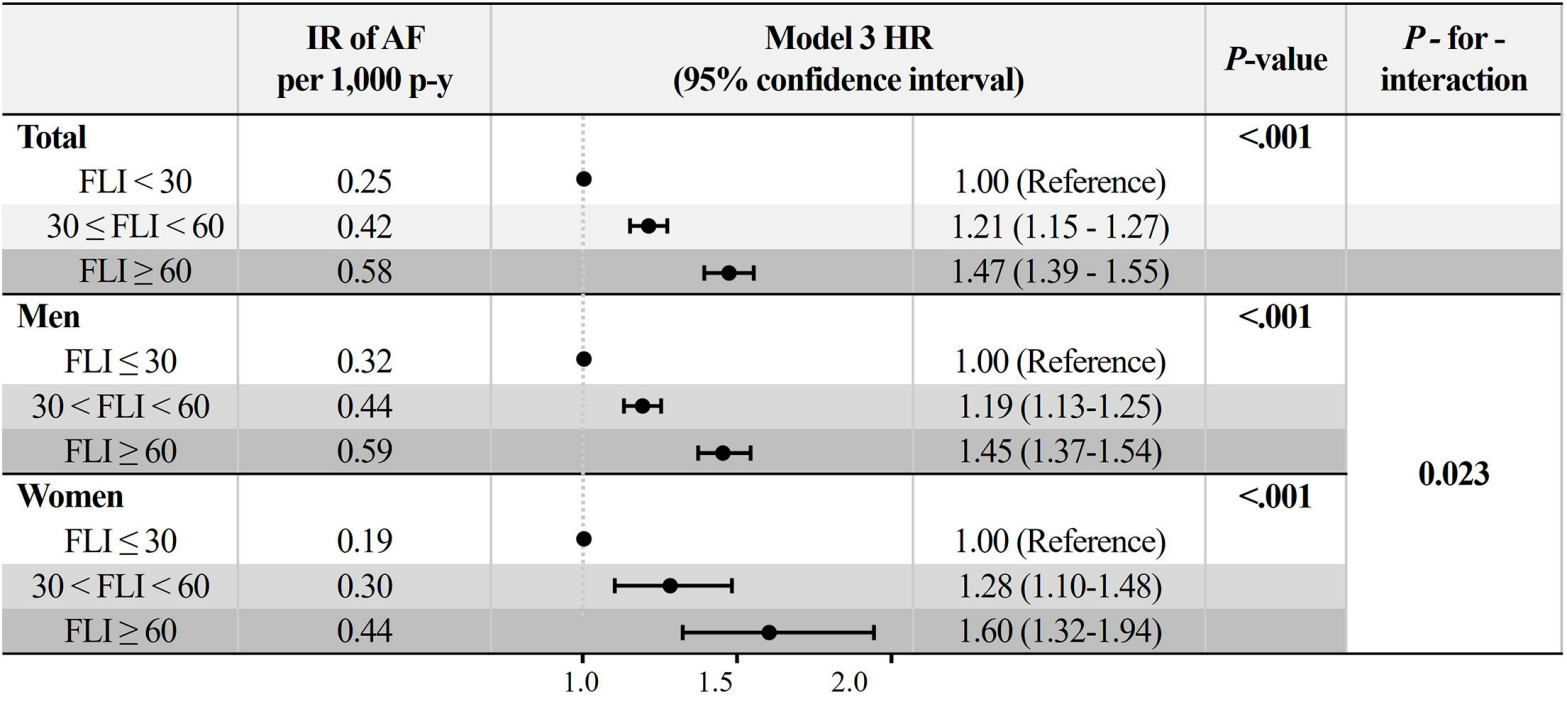
Incidence rate and adjusted hazard ratios (95% confidence interval) of AF in different fatty liver index group. Model 3: adjustment of age, sex, hypertension, diabetes mellitus, dyslipidemia, heart failure, prior ischemic stroke, prior myocardial infarction, chronic obstructive pulmonary disease, chronic kidney disease, sleep apnea, hyperthyroidism, smoking, alcohol consumption, and low income. AF, atrial fibrillation; IR, incidence rate.

**Figure 3:**
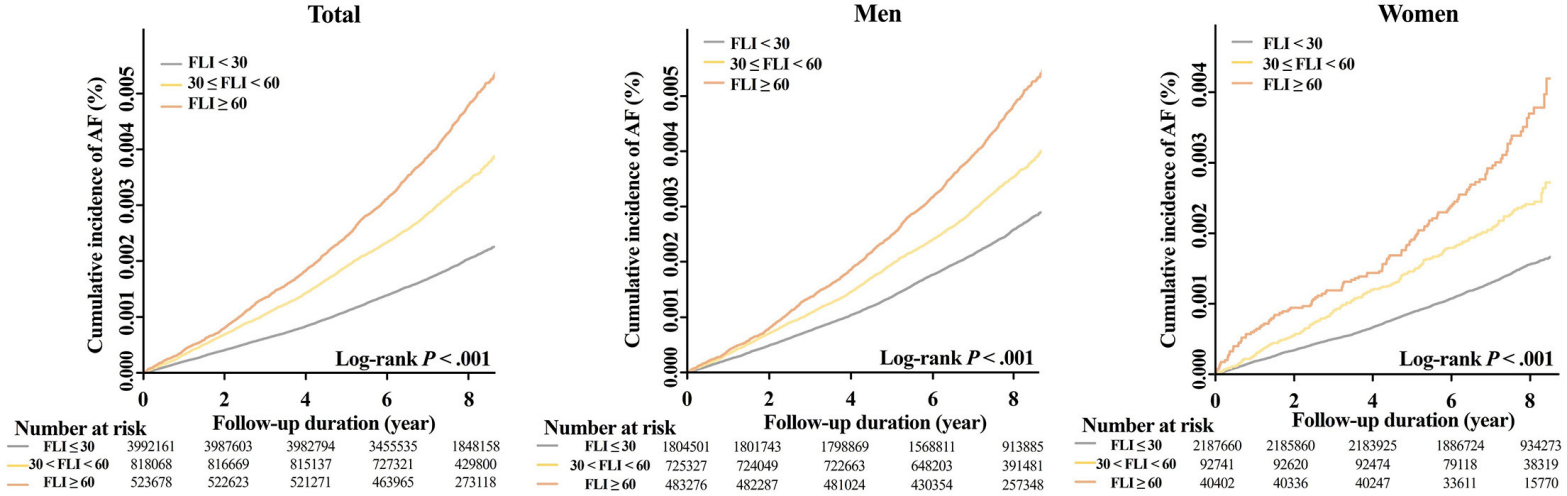
Cumulative incidence curves of AF stratified by FLI group in total, men, and women. AF, atrial fibrillation; FLI, fatty liver index.

During a mean follow-up duration of 7.4 ± 1.1 years, 12,096 subjects were newly diagnosed with AF (IR 0.31 per 1,000 person-years). The crude IR of AF increased in subjects with a higher FLI: 0.25 for the FLI <30 group, 0.42 for FLI 30 to <60, and 0.58 for FLI ≥ 60 groups, respectively (*P* for trends < 0.001, Figure 2). The cumulative incidence curves for AF according to different FLI ranges are shown in Figure 3.

After multivariable adjustment by model 3, compared to subjects with FLI <30, those with higher FLI were associated with a higher risk of incident AF (FLI 30 to <60 group: HR 1.21, 95% CI 1.15 to 1.27, and FLI≥60 group: HR 1.47, 95% CI 1.39 to 1.55) (Figure 2 and Supplementary Table 2). The increased risk of AF in subjects with higher FLI was slightly accentuated in women than in men (*P*-for-interaction = 0.023, Figure 2).

### FLI in a Decile Group

**Figure 4:**
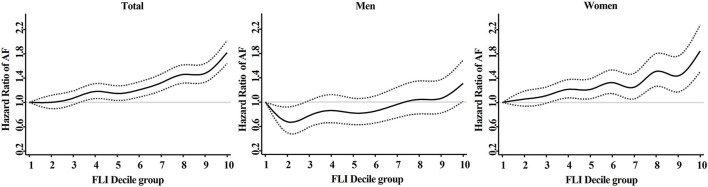
Adjusted hazard ratios (95% confidence interval) of AF in different fatty liver index decile group. Model 3: adjustment of age, sex, hypertension, diabetes mellitus, dyslipidemia, heart failure, prior ischemic stroke, prior myocardial infarction, chronic obstructive pulmonary disease, chronic kidney disease, sleep apnea, hyperthyroidism, smoking, alcohol consumption, and low income. AF, atrial fibrillation; FLI, fatty liver index.

The adjusted HR of FLI in decile groups are described in Figure 4. The adjusted HR showed consistently increasing trend in the total population (*P* < 0.001) and in subgroup of women (*P* < 0.001). In subgroup of men, the adjusted HR decreased to 0.67 (95% CI 0.49 to 0.92) at decile group 2. More detailed adjusted HRs are presented at Supplementary Table 3.

### Subgroup Analyses

**Figure 5:**
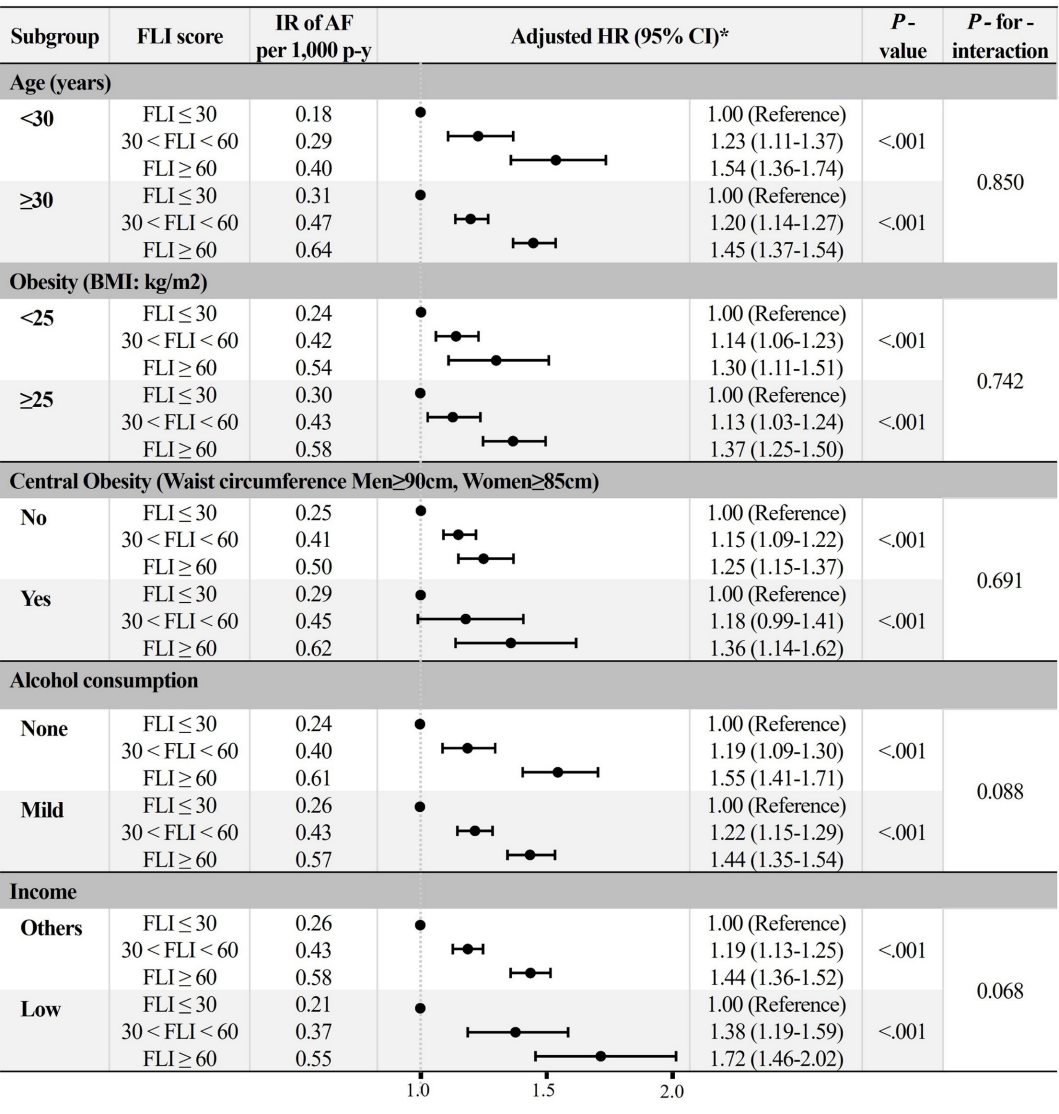
Subgroup analyses. *Adjusted HR: Model 3 (age, sex, hypertension, diabetes mellitus, dyslipidemia, heart failure, prior ischemic stroke, prior myocardial infarction, chronic obstructive pulmonary disease, chronic kidney disease, sleep apnea, hyperthyroidism, smoking, alcohol consumption, and low income). AF, atrial fibrillation; BMI, body mass index; FLI, fatty liver index; HR, hazard ratio; IR, incidence rate.

All subgroups showed a consistent trend of increased risk of AF in the higher FLI group after adjustment for Model 3 (Figure 5). *P*-for-interaction was insignificant in all subgroups, except for sex.

### Sensitivity Analysis

In those without significant cardiovascular comorbidities, the sensitivity analysis for an apparently healthy population showed findings consistent with the main results.

In an apparently healthy population, subjects with higher FLI were associated with an increased risk of incident AF compared to those with FLI <30 (FLI 30 to <60 group: HR 1.23, 95% CI 1.16 to 1.30, FLI ≥60 group: HR 1.56, 95% CI 1.45 1.66) (Table 2).

**Table 2:** Sensitivity analyses: analyses on subjects without significant cardiovascular comorbidities including hypertension, diabetes mellitus, dyslipidaemia, heart failure, prior ischemic stroke, and prior myocardial infarction.

	**FLI score**	**Number**	**Event**	**IR**	**Model 3** **HR (95% CI)**	***P*** **-value**
Total	FLI <30	3,714,063	6,356	0.23	1.00 (reference)	<0.001
	30 ≤ FLI <60	627,307	1,785	0.38	1.23 (1.16–1.30)	
	FLI ≥ 60	297,746	1,093	0.49	1.56 (1.45–1.66)	
Men	FLI <30	1,636,994	3,600	0.30	1.00 (reference)	<0.001
	30 ≤ FLI <60	556,890	1,655	0.40	1.21 (1.14–1.29)	
	FLI ≥ 60	273,866	1,030	0.50	1.53 (1.42–1.64)	
Women	FLI <30	2,077,069	2,756	0.18	1.00 (reference)	<0.001
	30 ≤ FLI <60	70,417	130	0.26	1.27 (1.06–1.51)	
	FLI ≥ 60	23,880	63	0.37	1.88 (1.46–2.41)	

*IR, per 1,000 person-years*.

*Model 3: age, sex, smoking, alcohol consumption, regular exercise, diabetes mellitus, hypertension, dyslipidaemia, heart failure, prior ischemic stroke, prior myocardial infarction, chronic obstructive pulmonary disease, chronic kidney disease, sleep apnea, hyperthyroidism, and low income*.

*CI, confidence interval; DM, diabetes mellitus; DL, dyslipidaemia; FLI, fatty liver index; HF, heart failure; HR, hazard ratio; HTN, hypertension; IR, incidence rate; MI, myocardial infarction*.

## Discussion

In this large-scale nationwide population-based cohort study, our main findings were as follows: (1) young adults with higher FLI had a higher risk of incident AF; (2) a positive correlation between FLI and the risk of AF was consistently observed in various subgroups without significant interaction except for sex; (3) increased risk of AF in subjects with higher FLI was more accentuated in women than in men; and (4) the association between higher FLI and increased risk of AF was consistently observed in apparently healthy subjects without significant cardiovascular comorbidities.

To the best of our knowledge, this is the first study to assess the risk of incident AF in young adults using FLI in a large population-based study.

In previous studies, the association between NAFLD and the risk of AF has been reported in selected patients with comorbidities or age of middle to elderly or those with definite ICD-10 code diagnosis of NAFLD (13, 15, 34). For example, Targher et al. have shown ultrasonography diagnosed NAFLD to be associated with an increased odds ratio of AF over 5 folds in patients with type 2 diabetes (13, 14). The Oulu Project Elucidating Risk of Atherosclerosis (OPERA) study showed that the risk of AF increased up to ~2-fold with ultrasonography diagnosed NAFLD in middle-aged hypertensive subjects (15). Furthermore, Labenz et al. reported that NAFLD, as defined by ICD-10 code, was significantly associated with the incidence of AF in both patients with age 18–40 years and 41–60 years in the German population with HR of 2.98 (95% CI 1.22 to 7.93) and 1.48 (95% CI 1.21 to 1.81), respectively (34). Regarding Korean population, Roh et al. previously reported an increased risk of AF up to 1.55 (95% CI 1.19 to 2.04) in a higher FLI quartile group in the Korean population (17). However, these studies did not precisely compare the association between FLI and the risk of AF in the young age group.

Although the prevalence of NAFLD is increasing by aging, NAFLD is common even in young adults (22.4% in the 30s vs. 34.0% in the 70s) (35). In AF, aging is the most potent risk factor with relatively low prevalence in young adults (1). The clinical impact of AF could be greater in young adults. AF is not easily curable, significantly affects patients' quality of life, and is associated with increased risks of cognitive dysfunction, stroke, heart failure, and all-cause death. The relative risks of AF-related complications are generally more prominent in young patients. However, the risk factors and substrate abnormalities for AF in young adults were not well-defined (36). Looking into the risk factor for AF by different age groups, the impact of metabolic factors such as diabetes mellitus or abdominal obesity on the risk of AF was more prominent in young adults aged 20 to <40 years (6). Identifying risk factors for AF in young adults and managing these properly might be needed to reduce the risk of AF.

It is still uncertain whether NAFLD is a risk factor for AF alone or simply reflects associated cardiovascular disease. However, the association between FLI and increased risk of AF might be due to FLI being a surrogate marker of metabolic unhealthiness. Previous studies have shown an increased risk of AF in metabolically unhealthy subjects with increased central obesity and BMI (7–10). As such components are also used in FLI, the correlation shown might simply be due to the reflection of the metabolic unhealthiness of the individual. This metabolic unhealthiness represented as FLI was associated with AF incident regardless of the amount of alcohol consumption (none or mild) in this study. With a recent update in the nomenclature NAFLD to metabolic dysfunction-associated fatty liver disease (MAFLD) (37), the metabolic unhealthiness has gained more emphasis. The diagnostic criteria of MAFLD includes BMI, TG, and waist circumference which are main components of FLI. FLI might help identifying those at higher risk of incident AF among the heterogeneous population of MAFLD.

Because increased FLI served as a surrogate marker for insulin resistance, insulin resistance occurred both atrial structural remodeling and abnormal intracellular calcium homeostasis, contributing to increased AF susceptibility (38–41). In a recent study (40, 41), insulin resistance-induced animals had a more susceptible atrium than the controls, with abnormal diastolic calcium leak and repetitive ectopic focal discharge in isolated atrial myocytes and whole atria, implying that calcium-related triggered activity as the mechanism of AF. Additionally, rats with insulin resistance demonstrated increased oxidative stress, NADPH oxidase activity, and CaMKII oxidation, as well as calcium handling- and structural remodeling processes.

Systemic inflammation may be an important mediator of pathophysiological links between NAFLD and AF. Those with NAFLD have increased accumulation of TG in the liver, increased free fatty acid, and increased production of reactive oxidative species, resulting in activation of the inflammatory response (42, 43). Previous studies have shown that increased low-grade, long-standing systemic inflammation in the liver leads to extrahepatic disease (44). For example, tumor necrosis factor α is one of the pro-inflammatory cytokines increased in the NAFLD model of mouse (45) and is associated with AF (46, 47). Thus, FLI, a surrogate marker of NAFLD, might reflect the systemic pro-inflammation state in NAFLD, leading to incident AF.

In this study, the FLI group showed an increased risk of AF along with an increase in the FLI group even at 30 to <60, which is not considered to be NAFLD in the “usual” cut-off value of 60. This trend was consistent in subgroup analyses of age, sex, obesity, central obesity, alcohol consumption, and income, which showed a consistent increase in the risk of AF along with higher FLI. This consistency may be evidence for lifestyle modification targeted at controlling factors even in subjects who are considered to be at risk of NAFLD and those who are diagnosed with NAFLD. Indeed, lifestyle modification is part of the integrated approach to AF characterization and treatment (48, 49). Such an integrated approach to AF management has been associated with improved clinical outcomes (50, 51).

Generally, NAFLD is more prevalent and severe in men than in women throughout reproductive age (52–54). Consistent with this, male predominancy was observed in subgroups with FLI ≥60 in the present analysis. The sex difference in the prevalence of NAFLD might be caused by the difference of tissue vulnerability on the proinflammatory/profibrotic cytokines, the effect of sex hormone, or the differences in the comorbid condition such as obesity, insulin resistance, and dyslipidemia. Although there was an uneven distribution of sexes within each FLI group, there was a statistically significant association between higher FLI and increased risk of AF in both men and women subgroups. In the present study, the risk of incident AF according to higher FLI values was slightly accentuated in women (*P*-for-interaction = 0.023).

Although the mechanism cannot be fully explained, there are possible hypothesis for the accentuation in women. The increased NAFLD is associated with increased epicardial adipose tissue and pericardial adipose tissue (PAT), (55–57) but the effect of PAT differs according to sex (58). PAT was more strongly associated with TG, impaired fasting glucose, and metabolic syndrome in women, (58) thus affecting the difference in the risks of incident AF between men and women.

### Limitations

This study had some limitations. First, the validation of NAFLD diagnosis could not be performed due to the absence of information on ultrasonography or histologic data. However, the external validation of FLI has already shown an area under receiver operating characteristics (AUROC) curve of 0.807 (59). In the Korean population, the AUROC of FLI was found to be 0.785 (60). Second, those with undetected asymptomatic AF could be underdiagnosed leading to underestimation of this arrhythmia (4, 61, 62). Third, unmeasured confounders including echocardiographic data and values which reflect inflammatory status, such as C-reactive protein (63), tumor necrosis factor (46), and interleukin-6 (64), could not be adjusted for, as they are not regularly checked in a health examination. In addition, although the prevalence and incidence of significant heart valve disease might be relatively low in this young population, the presence of valvular heart diseases might affect the risk of AF. We could not evaluate the genetic predisposition for AF in this population due to the inherent limitation of the data source. Fifth, we included variables for the multivariable Cox analysis that showed significant differences among groups in baseline characteristics. These selected comorbidities and lifestyle factors were well-known independent risk factors for AF or recently validated from the Korean nationwide population dataset (6, 65). There is a possibility of the presence of interactions among different variables. Sixth, we applied the same FLI cut-off for men and women in this analysis. In a previous report, different FLI cut-offs for men and women would be suggested to predict NAFLD (66). Instead of applying different cut-off in different sexes, we complementarily provided the results of FLI decile (in total population: FLI < 1.94, FLI < 3.04, FLI < 4.53, FLI < 6.74, FLI < 10.16, FLI < 15.56, FLI < 24.16, FLI < 37.82, FLI < 59.51, 59.51 ≤ FLI) and the risk of AF in both men and women. Similarly, although the cut-off value for alcohol consumption differs between different sexes, we used a single cut-off of 30 for both sexes. This might have affected the increased risk of AF in women. Lastly, our study was analyzed in a homogenous Korean population and cannot be directly generalized to other ethnicities.

## Conclusions

Among young adults, NAFLD assessed using FLI was positively correlated with the risk of AF. These findings support the evidence of AF screening in young adults with high FLI scores.

## Data Availability Statement

Publicly available datasets were analyzed in this study. This data can be found here: https://nhiss.nhis.or.kr/bd/ab/bdaba021eng.do, Korean National Health Insurance Service.

## Ethics Statement

The studies involving human participants were reviewed and approved by Seoul National University Hospital Institutional Review Board. Written informed consent for participation was not required for this study in accordance with the national legislation and the institutional requirements.

## Author Contributions

This study was coordinated by E-KC as the principal investigator. S-WL and K-DH went over the statistics. JCho and S-RL contributed to writing and original draft preparation with support from H-JA, SK, S-HP, HL, JChu, and MH. SO and GL supervised the findings of this work. All authors contributed to the article and approved the submitted version.

## Funding

This work was supported in part by the Korea Medical Device Development Fund grant funded by the Korean government (the Ministry of Science and ICT, the Ministry of Trade, Industry and Energy, the Ministry of Health & Welfare, and the Ministry of Food and Drug Safety) (Project Numbers: HI20C1662, 1711138358, and KMDF_PR_20200901_0173) and by the Korea National Research Foundation funded by the Ministry of Education, Science and Technology (Grant 2020R1F1A106740).

## Conflict of Interest

E-KC, Research grants or speaking fees from Bayer, BMS/Pfizer, Biosense Webster, Chong Kun Dang, Daiichi-Sankyo, Dreamtech Co., Ltd., Medtronic, Samjinpharm, Sanofi-Aventis, Seers Technology, and Skylabs. GL, Consultant and speaker for BMS/Pfizer, Boehringer Ingelheim, and Daiichi-Sankyo. The remaining authors declare that the research was conducted in the absence of any commercial or financial relationships that could be construed as a potential conflict of interest.

## Publisher's Note

All claims expressed in this article are solely those of the authors and do not necessarily represent those of their affiliated organizations, or those of the publisher, the editors and the reviewers. Any product that may be evaluated in this article, or claim that may be made by its manufacturer, is not guaranteed or endorsed by the publisher.

## References

[1] Lee SR Choi EK Han KD Cha MJ Oh S. Trends in the incidence and prevalence of atrial fibrillation and estimated thromboembolic risk using the CHA(2)DS(2)-VASc score in the entire Korean population. Int J Cardiol. (2017) 236:226–31. 10.1016/j.ijcard.2017.02.039 28233629

[2] Burdett P Lip GYH. Atrial Fibrillation in the United Kingdom: predicting costs of an emerging epidemic recognising and forecasting the cost drivers of atrial fibrillation-related costs. Eur Heart J Qual Care Clin Outcomes. (2020) 8:187–94. 10.1093/ehjqcco/qcaa093 33346822

[3] Lee E Choi EK Han KD Lee H Choe WS Lee SR. Mortality and causes of death in patients with atrial fibrillation: A nationwide population-based study. PLoS One. (2018) 13:e0209687. 10.1371/journal.pone.0209687 30586468 PMC6306259

[4] Wallenhorst C Martinez C Freedman B. Risk of ischemic stroke in asymptomatic atrial fibrillation incidentally detected in primary care compared with other clinical presentations. Thromb Haemost. (2022) 122:277–85. 10.1055/a-1541-3885 34192776

[5] Kivioja R Pietilä A Martinez-Majander N Gordin D Havulinna AS Salomaa V. Risk factors for early-onset ischemic stroke: a case-control study. J Am Heart Assoc. (2018) 7:e009774. 10.1161/JAHA.118.009774 30608196 PMC6404210

[6] Kim YG Han KD Choi JI Choi YY Choi HY Boo KY. Non-genetic risk factors for atrial fibrillation are equally important in both young and old age: a nationwide population-based study. Eur J Prev Cardiol. (2021) 28:666–76. 10.1177/2047487320915664 34021574

[7] Ahn HJ Han KD Choi EK Jung JH Kwon S Lee SR. Cumulative burden of metabolic syndrome and its components on the risk of atrial fibrillation: a nationwide population-based study. Cardiovasc Diabetol. (2021) 20:20. 10.1186/s12933-021-01215-8 33468142 PMC7816376

[8] Nyström PK Carlsson AC Leander K de Faire U Hellenius ML Gigante B. Obesity, metabolic syndrome and risk of atrial fibrillation: a Swedish, prospective cohort study. PLoS One. (2015) 10:e0127111. 10.1371/journal.pone.0127111 25978738 PMC4433194

[9] Kim YG Choi KJ Han S Hwang KW Kwon CH Park GM. Metabolic syndrome and the risk of new-onset atrial fibrillation in middle-aged East Asian men. Circ J. (2018) 82:1763–9. 10.1253/circj.CJ-18-0113 29743419

[10] Fauchier G Bisson A Bodin A Herbert J Semaan C Angoulvant D. Metabolically healthy obesity and cardiovascular events: a nationwide cohort study. Diabetes Obes Metab. (2021) 23:2492–501. 10.1111/dom.14492 34251088

[11] Wong W-K Chan W-K. Nonalcoholic fatty liver disease: a global perspective. clinical therapeutics. (2021) 43:473–99. 10.1016/j.clinthera.2021.01.007 33526312

[12] Kang SY Kim YJ Park HS. Trends in the prevalence of non-alcoholic fatty liver disease and its future predictions in Korean men, 1998–2035. J Clin Med. (2020) 9:2626. 10.3390/jcm9082626 32823604 PMC7465994

[13] Targher G Valbusa F Bonapace S Bertolini L Zenari L Rodella S. Non-alcoholic fatty liver disease is associated with an increased incidence of atrial fibrillation in patients with type 2 diabetes. PLoS ONE. (2013) 8:e57183. 10.1371/journal.pone.0057183 23451184 PMC3579814

[14] Targher G Mantovani A Pichiri I Rigolon R Dauriz M Zoppini G. Non-alcoholic fatty liver disease is associated with an increased prevalence of atrial fibrillation in hospitalized patients with type 2 diabetes. Clin Sci. (2013) 125:301–9. 10.1042/CS20130036 23596966

[15] Käräjämäki AJ Pätsi O-P Savolainen M Kesäniemi YA Huikuri H Ukkola O. Non-alcoholic fatty liver disease as a predictor of atrial fibrillation in middle-aged population (OPERA Study). PLoS One. (2015) 10:e0142937. 10.1371/journal.pone.0142937 26571029 PMC4646339

[16] Bedogni G Bellentani S Miglioli L Masutti F Passalacqua M Castiglione A. The fatty liver index: a simple and accurate predictor of hepatic steatosis in the general population. BMC Gastroenterol. (2006) 6:33. 10.1186/1471-230X-6-33 17081293 PMC1636651

[17] Roh J-H Lee J-H Lee H Yoon Y-H Kim M Kim Y-G. Association between non-alcoholic fatty liver disease and risk of new-onset atrial fibrillation in healthy adults. Liver Int. (2020) 40:338–46. 10.1111/liv.14236 31479572

[18] Huang X Xu M Chen Y Peng K Huang Y Wang P. Validation of the fatty liver index for nonalcoholic fatty liver disease in middle-aged and elderly Chinese. Medicine. (2015) 94:e1682-e. 10.1097/MD.0000000000001682 26448014 PMC4616754

[19] Lee SR Han KD Choi EK Oh S Lip GYH. Nonalcoholic fatty liver disease and the risk of atrial fibrillation stratified by body mass index: a nationwide population-based study. Sci Rep. (2021) 11:3737. 10.1038/s41598-021-83367-x 33580177 PMC7881181

[20] NHIS. National Health Screening Statistical Yearbook: National Health Insurance Service. Available online at: https://www.nhis.or.kr/nhis/together/wbhaec07000m01.do?mode=view&articleNo=10806637&article.offset=0&articleLimit=10. (accessed September 22, 2021).

[21] Cheol Seong S Kim Y-Y Khang Y-H Heon Park J Kang H-J Lee H. Data resource profile: the national health information database of the national health insurance service in South Korea. Int J Epidemiol. (2016) 46:799–800. 10.1093/ije/dyw253 27794523 PMC5837262

[22] Choi EK. Cardiovascular research using the korean national health information database. Korean Circ J. (2020) 50:754–72. 10.4070/kcj.2020.0171 32725984 PMC7441000

[23] Sanyal AJ. AGA technical review on nonalcoholic fatty liver disease. Gastroenterology. (2002) 123:1705–25. 10.1053/gast.2002.36572 12404245

[24] Chalasani N Younossi Z Lavine JE Diehl AM Brunt EM Cusi K. The diagnosis and management of non-alcoholic fatty liver disease: practice Guideline by the American Association for the Study of Liver Diseases, American College of Gastroenterology, and the American Gastroenterological Association. Hepatology. (2012) 55:2005–23. 10.1002/hep.25762 22488764

[25] Hernaez R Lazo M Bonekamp S Kamel I Brancati FL Guallar E. Diagnostic accuracy and reliability of ultrasonography for the detection of fatty liver: a meta-analysis. Hepatology. (2011) 54:1082–90. 10.1002/hep.24452 21618575 PMC4197002

[26] Huh JH Kim JY Choi E Kim JS Chang Y Sung K-C. The fatty liver index as a predictor of incident chronic kidney disease in a 10-year prospective cohort study. PLoS One. (2017) 12:e0180951. 10.1371/journal.pone.0180951 28738057 PMC5524328

[27] Jäger S Jacobs S Kröger J Stefan N Fritsche A Weikert C. Association between the fatty liver index and risk of type 2 diabetes in the EPIC-potsdam study. PLoS One. (2015) 10:e0124749. 10.1371/journal.pone.0124749 25902304 PMC4406732

[28] Chung TH Kim JK Kim JH Lee YJ. Fatty liver index as a simple and useful predictor for 10-year cardiovascular disease risks determined by framingham risk score in the general Korean population. J Gastrointestin Liver Dis. (2021) 30:221–6. 10.15403/jgld-3404 34174059

[29] Bansal N Zelnick LR Alonso A Benjamin EJ de Boer IH Deo R. eGFR and albuminuria in relation to risk of incident atrial fibrillation: a meta-analysis of the jackson heart study, the multi-ethnic study of atherosclerosis, and the cardiovascular health study. Clin J Am Soc Nephrol. (2017) 12:1386–98. 10.2215/CJN.01860217 28798221 PMC5586568

[30] Lee SR Choi EK Han KD Lee SH Oh S. Effect of the variability of blood pressure, glucose level, total cholesterol level, and body mass index on the risk of atrial fibrillation in a healthy population. Heart Rhythm. (2020) 17:12–9. 10.1016/j.hrthm.2019.07.006 31299298

[31] Park CS Han KD Choi EK Kim DH Lee HJ Lee SR. Lifestyle is associated with atrial fibrillation development in patients with type 2 diabetes mellitus. Sci Rep. (2021) 11:4676. 10.1038/s41598-021-84307-5 33633333 PMC7907194

[32] Lee SR Choi EK Ahn HJ Han KD Oh S Lip GYH. Association between clustering of unhealthy lifestyle factors and risk of new-onset atrial fibrillation: a nationwide population-based study. Sci Rep. (2020) 10:19224. 10.1038/s41598-020-75822-y 33154443 PMC7645499

[33] Lee SS Ae Kong K Kim D Lim Y-M Yang P-S Yi J-E. Clinical implication of an impaired fasting glucose and prehypertension related to new onset atrial fibrillation in a healthy Asian population without underlying disease: a nationwide cohort study in Korea. Eur Heart J. (2017) 38:2599–607. 10.1093/eurheartj/ehx316 28662568

[34] Labenz C Huber Y Michel M Nagel M Galle PR Kostev K. Impact of NAFLD on the incidence of cardiovascular diseases in a primary care population in Germany. Dig Dis Sci. (2020) 65:2112–9. 10.1007/s10620-019-05986-9 31797186

[35] Younossi ZM Koenig AB Abdelatif D Fazel Y Henry L Wymer M. Global epidemiology of nonalcoholic fatty liver disease-Meta-analytic assessment of prevalence, incidence, and outcomes. Hepatology. (2016) 64:73–84. 10.1002/hep.28431 26707365

[36] Sankaranarayanan R Kirkwood G Dibb K Garratt CJ. Comparison of atrial fibrillation in the young versus that in the elderly: a review. Cardiol Res Pract. (2013) 2013:976976. 10.1155/2013/976976 23401843 PMC3564268

[37] Eslam M Newsome PN Sarin SK Anstee QM Targher G Romero-Gomez M. A new definition for metabolic dysfunction-associated fatty liver disease: an international expert consensus statement. J Hepatol. (2020) 73:202–9. 10.1016/j.jhep.2020.03.039 32278004

[38] Watanabe M Yokoshiki H Mitsuyama H Mizukami K Ono T Tsutsui H. Conduction and refractory disorders in the diabetic atrium. Am J Physiol Heart Circ Physiol. (2012) 303:H86–95. 10.1152/ajpheart.00010.2012 22561303

[39] Hohl M Lau DH Müller A Elliott AD Linz B Mahajan R. Concomitant obesity and metabolic syndrome add to the atrial arrhythmogenic phenotype in male hypertensive rats. J Am Heart Assoc. (2017) 6:e006717. 10.1161/JAHA.117.006717 28919580 PMC5634308

[40] Axelsen LN Calloe K Braunstein TH Riemann M Hofgaard JP Liang B. Diet-induced pre-diabetes slows cardiac conductance and promotes arrhythmogenesis. Cardiovasc Diabetol. (2015) 14:87. 10.1186/s12933-015-0246-8 26169175 PMC4504126

[41] Chan YH Chang GJ Lai YJ Chen WJ Chang SH Hung LM. Atrial fibrillation and its arrhythmogenesis associated with insulin resistance. Cardiovasc Diabetol. (2019) 18:125. 10.1186/s12933-019-0928-8 31558158 PMC6761716

[42] Chen Z Tian R She Z Cai J Li H. Role of oxidative stress in the pathogenesis of nonalcoholic fatty liver disease. Free Radic Biol Med. (2020) 152:116–41. 10.1016/j.freeradbiomed.2020.02.025 32156524

[43] Kawano Y Cohen DE. Mechanisms of hepatic triglyceride accumulation in non-alcoholic fatty liver disease. J Gastroenterol. (2013) 48:434–41. 10.1007/s00535-013-0758-5 23397118 PMC3633701

[44] Adams LA Anstee QM Tilg H Targher G. Non-alcoholic fatty liver disease and its relationship with cardiovascular disease and other extrahepatic diseases. Gut. (2017) 66:1138–53. 10.1136/gutjnl-2017-313884 28314735

[45] Wandrer F Liebig S Marhenke S Vogel A John K Manns MP. TNF-Receptor-1 inhibition reduces liver steatosis, hepatocellular injury and fibrosis in NAFLD mice. Cell Death Dis. (2020) 11:212. 10.1038/s41419-020-2411-6 32235829 PMC7109108

[46] Li J Solus J Chen Q Rho YH Milne G Stein CM. Role of inflammation and oxidative stress in atrial fibrillation. Heart Rhythm. (2010) 7:438–44. 10.1016/j.hrthm.2009.12.009 20153266 PMC2843774

[47] Deng H Xue YM Zhan XZ Liao HT Guo HM Wu SL. Role of tumor necrosis factor-alpha in the pathogenesis of a trial fibrillation. Chin Med J. (2011) 124:1976–82. 10.3760/cma.j.issn.0366-6999.2011.13.010 22088456

[48] Potpara TS Lip GYH Blomstrom-Lundqvist C Boriani G van Gelder IC Heidbuchel H. The 4S-AF Scheme (stroke risk; symptoms; severity of burden; substrate): a novel approach to in-depth characterization (rather than classification) of atrial fibrillation. Thromb Haemost. (2021) 121:270–8. 10.1055/s-0040-1716408 32838473

[49] Lip GYH. The ABC pathway: an integrated approach to improve AF management. Nat Rev Cardiol. (2017) 14:627–8. 10.1038/nrcardio.2017.153 28960189

[50] Yoon M Yang PS Jang E Yu HT Kim TH Uhm JS. Improved population-based clinical outcomes of patients with atrial fibrillation by compliance with the simple ABC (Atrial Fibrillation Better Care) pathway for integrated care management: a nationwide cohort study. Thromb Haemost. (2019) 119:1695–703. 10.1055/s-0039-1693516 31266082

[51] Romiti GF Pastori D Rivera-Caravaca JM Ding WY Gue YX Menichelli D. Adherence to the 'atrial fibrillation better care' pathway in patients with atrial fibrillation: impact on clinical outcomes-a systematic review and meta-analysis of 285,000 patients. Thromb Haemost. (2021). 10.1055/a-1515-9630. [Epub ahead of print]. 34020488

[52] Lonardo A Nascimbeni F Ballestri S Fairweather D Win S Than TA. Sex differences in nonalcoholic fatty liver disease: state of the art and identification of research gaps. Hepatology. (2019) 70:1457–69. 10.1002/hep.30626 30924946 PMC6766425

[53] Lazo M Hernaez R Eberhardt MS Bonekamp S Kamel I Guallar E. Prevalence of nonalcoholic fatty liver disease in the United States: the third national health and nutrition examination survey, 1988–1994. Am J Epidemiol. (2013) 178:38–45. 10.1093/aje/kws448 23703888 PMC3698993

[54] Caballería L Pera G Arteaga I Rodríguez L Alumà A Morillas RM. High prevalence of liver fibrosis among European adults with unknown liver disease: a population-based study. Clin Gastroenterol Hepatol. (2018) 16:1138–45.e5. 10.1016/j.cgh.2017.12.048 29452268

[55] Brouha SS Nguyen P Bettencourt R Sirlin CB Loomba R. Increased severity of liver fat content and liver fibrosis in non-alcoholic fatty liver disease correlate with epicardial fat volume in type 2 diabetes: a prospective study. Eur Radiol. (2018) 28:1345–55. 10.1007/s00330-017-5075-6 29058029 PMC6310479

[56] Liu B Li Y Li Y Liu Y Yan Y Luo A. Association of epicardial adipose tissue with non-alcoholic fatty liver disease: a meta-analysis. Hepatol Int. (2019) 13:757–65. 10.1007/s12072-019-09972-1 31432447

[57] Ni X Jiao L Zhang Y Xu J Zhang Y Zhang X. Relationship between non-alcoholic fatty liver disease and abdominal and pericardial adipose tissue in middle-aged and elderly subjects. Int J Gen Med. (2021) 14:3439–44. 10.2147/IJGM.S317081 34285567 PMC8286728

[58] Rosito GA Massaro JM Hoffmann U Ruberg FL Mahabadi AA Vasan RS. Pericardial fat, visceral abdominal fat, cardiovascular disease risk factors, and vascular calcification in a community-based sample: the Framingham heart study. Circulation. (2008) 117:605–13. 10.1161/CIRCULATIONAHA.107.743062 18212276

[59] Koehler EM Schouten JN Hansen BE Hofman A Stricker BH Janssen HL. External validation of the fatty liver index for identifying nonalcoholic fatty liver disease in a population-based study. Clin Gastroenterol Hepatol. (2013) 11:1201–4. 10.1016/j.cgh.2012.12.031 23353640

[60] Kim JH Kwon SY Lee SW Lee CH. Validation of fatty liver index and lipid accumulation product for predicting fatty liver in Korean population. Liver Int. (2011) 31:1600–1. 10.1111/j.1478-3231.2011.02580.x 22093336

[61] Kitsiou A Rogalewski A Kalyani M Deelawar S Tribunyan S Greeve I. Atrial fibrillation in patients with embolic stroke of undetermined source during 3 years of prolonged monitoring with an implantable loop recorder. Thromb Haemost. (2021) 121:826–33. 10.1055/a-1346-2899 33401327

[62] Sun W Freedman B Martinez C Wallenhorst C Yan BP. Atrial fibrillation detected by single time-point handheld electrocardiogram screening and the risk of ischemic stroke. Thromb Haemost. (2022) 122:286–94. 10.1055/a-1588-8867 34399432

[63] Aviles RJ Martin DO Apperson-Hansen C Houghtaling PL Rautaharju P Kronmal RA. Inflammation as a risk factor for atrial fibrillation. Circulation. (2003) 108:3006–10. 10.1161/01.CIR.0000103131.70301.4F 14623805

[64] Marcus GM Whooley MA Glidden DV Pawlikowska L Zaroff JG Olgin JE. Interleukin-6 and atrial fibrillation in patients with coronary artery disease: data from the heart and soul study. Am Heart J. (2008) 155:303–9. 10.1016/j.ahj.2007.09.006 18215601 PMC2247366

[65] Lee SR Park CS Choi EK Ahn HJ Han KD Oh S. Hypertension burden and the risk of new-onset atrial fibrillation: a nationwide population-based study. Hypertension. (2021) 77:919–28. 10.1161/HYPERTENSIONAHA.120.16659 33486985

[66] Cho EJ Jung GC Kwak MS Yang JI Yim JY Yu SJ. Fatty liver index for predicting nonalcoholic fatty liver disease in an asymptomatic Korean population. Diagnostics. (2021) 11:2233. 10.3390/diagnostics11122233 34943469 PMC8699943

