# Supplementary material for: Non-alcoholic Fatty Liver Disease and the Risk of Incident Atrial Fibrillation in Young Adults: A Nationwide Population-Based Cohort Study
Source: Front Cardiovasc Med. 2022 Mar 23;9:832023. doi: 10.3389/fcvm.2022.832023 (PMC8984026; doi:10.3389/fcvm.2022.832023)
Supplement: Supplementary file 1 [file Table_1.DOCX]

Supplementary Material

**Supplementary Tables**

**Table S1.** Definitions of covariates

**Table S2.** Hazard ratios for atrial fibrillation according to the fatty liver index group of three

**Table S3**. Hazard ratios for atrial fibrillation according to the fatty liver index decile groups

**Supplementary Tables**

**Supplementary Table S1. Definitions of covariates**

| **Diagnosis** | **ICD-10-CM code and definition** | **Diagnostic definition** |
| --- | --- | --- |
| **Inclusion/exclusion criteria** |  |  |
| **Liver disease**  **Liver cirrhosis**  **Hepatitis**  **Hepatocellular carcinoma** | K703 and K76  B15–B19  C22 | Admission or outpatient department≥1 |
| **Atrial fibrillation** | I48.0-48.4, I48.9 | Admission or outpatient department≥1 |
| **Alcohol consumption**  **Heavy** | Alcohol consumption ≥30g per day | Index health examination |
| **Comorbidities** |  |  |
| **Hypertension** | I10-I13, I15; and minimum 1 prescription of anti-hypertensive drug (thiazide, loop diuretics, aldosterone antagonist, alpha-/beta-blocker, calcium-channel blocker, angiotensin-converting enzyme inhibitor, angiotensin II receptor blocker). | Admission≥1 or outpatient department≥1 |
|  | Or systolic/diastolic blood pressure ≥ 140/90 mmHg | Index health examination |
| **Diabetes mellitus** | E11-E14; and minimum 1 prescription of anti-diabetic drugs (sulfonylureas, metformin, meglitinides, thiazolidinediones, dipeptidyl peptidase-4 inhibitors, α-glucosidase inhibitors, and insulin). | Admission≥1 or outpatient department≥1 |
|  | Or fasting glucose level ≥ 126 mg/dL | Index health examination |
| **Dyslipidemia** | E78 | Admission or outpatient department≥1 |
|  | Or Total cholesterol ≥ 240 mg/dL | Index health examination |
| **Heart failure** | I50 | Admission or outpatient department≥1 |
| **Prior stroke** | I63, I64 | Admission or outpatient department≥1 |
| **Chronic kidney disease** | Estimated glomerular filtration rate <60 ml/min/1.73m^2^ | Index health examination |
| **Chronic obstructive pulmonary disease** | J41-44 | Admission or outpatient department≥1 |
| **Sleep apnea** | G473 | Admission or outpatient department≥1 |
| **Thyroid disease** | E05, E03 | Admission or outpatient department≥1 |
| **Health exam questionnaire** |  |  |
| **Smoking**  **Ex**  **Current** | Ex-smoker at the 1st examination and sustaining non-smoking till the 2nd examination  Current smoker at the 2nd examination regardless of the smoking status at the 1st examination. | Index health examination |
| **Alcohol consumption**  **Mild** | Alcohol consumption >0g to <30g per day | Index health examination |
| **Regular exercise** | Performing a moderate physical activity more than 30 minutes at least 5 times per week or strenuous physical activity more than 20 minutes at least 3 times per week. | Index health examination |
| **Low income** | Income lowest 20% among the entire Korean population and supported by the medical aid | Index health examination |

Abbreviations: ICD, international classification of disease; CM, clinical modification.

**Supplementary Table S2. Hazard ratios for atrial fibrillation according to the fatty liver index group of three**

| **FLI** | **Number** | **Event** | **IR**  **(per 1,000 PY)** | **Model 1**  **HR (95% CI)** | **Model 2**  **HR (95% CI)** | **Model 3**  **HR (95% CI)** |
| --- | --- | --- | --- | --- | --- | --- |
| **Total** | | | | | | |
| **FLI < 30** | 3,992,161 | 7264 | 0.25 | 1.00 (reference) | 1.00 (reference) | 1.00(reference) |
| **30 ≤ FLI < 60** | 818,068 | 2578 | 0.42 | 1.70 (1.62-1.78) | 1.271 (1.212,1.333) | 1.21 (1.153,1.27) |
| **FLI ≥ 60** | 523,678 | 2254 | 0.58 | 2.33 (2.23-2.45) | 1.691 (1.608,1.779) | 1.47 (1.392,1.552) |
| ***P* - value** | | | | <0.001 | <0.001 | <0.001 |
| **Men** | | | | | | |
| **FLI < 30** | 1,804,501 | 4250 | 0.32 | 1.00 (reference) | 1.00 (reference) | 1.00 (reference) |
| **30 ≤ FLI < 60** | 725,327 | 2376 | 0.44 | 1.37 (1.31-1.44) | 1.24 (1.18-1.30) | 1.19 (1.13-1.25) |
| **FLI ≥ 60** | 483,276 | 2128 | 0.59 | 1.86 (1.76-1.96) | 1.64 (1.56-1.73) | 1.45 (1.37-1.54) |
| ***P* - value** | | | | <0.001 | <0.001 | <0.001 |
| **Women** | | | | | | |
| **FLI < 30** | 2,187,660 | 3014 | 0.19 | 1.00 (reference) | 1.00(reference) | 1.00 (reference) |
| **30 ≤ FLI < 60** | 92,741 | 202 | 0.30 | 1.60 (1.39-1.85) | 1.46 (1.26-1.68) | 1.28 (1.10-1.48) |
| **FLI ≥ 60** | 40,402 | 126 | 0.44 | 2.33 (1.95-2.78) | 2.17 (1.82-2.59) | 1.60 (1.32-1.94) |
| ***P* - value** | | | | <0.001 | <0.001 | <0.001 |

Multivariable adjusted model included age, sex, hypertension, diabetes mellitus, dyslipidemia, heart failure, prior ischemic stroke, prior myocardial infarction, chronic obstructive pulmonary disease, chronic kidney disease, sleep apnea, hyperthyroidism, and low income.

Model 1: unadjusted

Model 2: age and sex

Model 3: age, sex, smoking, alcohol consumption, regular exercise, diabetes mellitus, Hypertension, dyslipidemia, heart failure, prior ischemic stroke, prior myocardial infarction, chronic obstructive pulmonary disease, chronic kidney disease, sleep apnea, hyperthyroidism, and low income.

Abbreviations: CI, confidence interval; FLI, fatty liver index; HR, hazard ratio; IR, incidence rate; PY, person-years.

**Supplementary Table S3. Hazard ratios for atrial fibrillation according to the fatty liver index decile groups**

| **FLI** | **Number** | **Event** | **IR**  **(per 1,000 PY)** | **Model 1**  **HR (95% CI)** | **Model 2**  **HR (95% CI)** | **Model 3**  **HR (95% CI)** |
| --- | --- | --- | --- | --- | --- | --- |
| **Total** | | | | | | |
| **D1 (<1.94)** | 533390 | 623 | 0.16 | 1.00 (reference) | 1.00 (reference) | 1.00(reference) |
| **D2 (<3.04)** | 533391 | 679 | 0.17 | 1.09 (0.98-1.21) | 1.00 (0.90-1.11) | 1.00 (0.90-1.12) |
| **D3 (<4.53)** | 533391 | 790 | 0.20 | 1.26 (1.13-1.40) | 1.07 (0.96-1.19) | 1.07 (0.96-1.19) |
| **D4 (<6.74)** | 533391 | 949 | 0.24 | 1.51 (1.36-1.67) | 1.19 (1.07-1.32) | 1.18 (1.06-1.31) |
| **D5 (<10.16)** | 533391 | 1003 | 0.26 | 1.59 (1.44-1.75) | 1.16 (1.04-1.29) | 1.15 (1.03-1.27) |
| **D6 (<15.56)** | 533390 | 1138 | 0.29 | 1.79 (1.62-1.97) | 1.23 (1.11-1.36) | 1.21 (1.09-1.34) |
| **D7 (<24.16)** | 533392 | 1336 | 0.34 | 2.09 (1.90-2.30) | 1.36 (1.23-1.51) | 1.32 (1.19-1.47) |
| **D8 (<37.82)** | 533390 | 1576 | 0.40 | 2.45 (2.24-2.69) | 1.53 (1.38-1.69) | 1.46 (1.32-1.62) |
| **D9 (<59.51)** | 533391 | 1703 | 0.43 | 2.65 (2.41-2.90) | 1.59 (1.43-1.76) | 1.48 (1.33-1.64) |
| **D10 (**≥**59.51)** | 533390 | 2299 | 0.58 | 3.59 (3.29-3.93) | 2.12 (1.91-2.34) | 1.82 (1.64-2.02) |
| ***P* - value** | | | | <0.001 | <0.001 | <0.001 |
| **Men** | | | | | | |
| **D1 (<1.94)** | 28072 | 61 | 0.30 | 1.00 (reference) | 1.00 (reference) | 1.00 (reference) |
| **D2 (<3.04)** | 75916 | 117 | 0.21 | 0.70 (0.52-0.96) | 0.68 (0.50-0.92) | 0.67 (0.49-0.92) |
| **D3 (<4.53)** | 144675 | 267 | 0.25 | 0.83 (0.63-1.10) | 0.78 (0.59-1.03) | 0.78 (0.59-1.02) |
| **D4 (<6.74)** | 228095 | 484 | 0.29 | 0.95 (0.73-1.24) | 0.87 (0.67-1.14) | 0.86 (0.66-1.12) |
| **D5 (<10.16)** | 309768 | 652 | 0.28 | 0.94 (0.72-1.22) | 0.83 (0.64-1.08) | 0.82 (0.63-1.06) |
| **D6 (<15.56)** | 376945 | 861 | 0.31 | 1.01 (0.78-1.31) | 0.87 (0.67-1.13) | 0.85 (0.66-1.10) |
| **D7 (<24.16)** | 423606 | 1147 | 0.36 | 1.19 (0.92-1.54) | 0.99 (0.77-1.28) | 0.96 (0.74-1.24) |
| **D8 (<37.82)** | 455796 | 1409 | 0.41 | 1.36 (1.05-1.75) | 1.09 (0.84-1.41) | 1.04 (0.81-1.35) |
| **D9 (<59.51)** | 478136 | 1584 | 0.44 | 1.45 (1.13-1.88) | 1.14 (0.88_1.47) | 1.06 (0.82-1.37) |
| **D10 (**≥**59.51)** | 492095 | 2172 | 0.59 | 1.95 (1.51-2.51) | 1.50 (1.16-1.94) | 1.31 (1.01-1.69) |
| ***P* - value** | | | | <0.001 | <0.001 | <0.001 |
| **Women** | | | | | | |
| **D1 (<1.94)** | 505318 | 562 | 0.15 | 1.00 (reference) | 1.00(reference) | 1.00 (reference) |
| **D2 (<3.04)** | 457475 | 562 | 0.17 | 1.10 (0.98-1.24) | 1.06 (0.94-1.19) | 1.06 (0.94-1.19) |
| **D3 (<4.53)** | 388716 | 523 | 0.18 | 1.21 (1.07-1.36) | 1.12 (0.99-1.26) | 1.11 (0.99-1.25) |
| **D4 (<6.74)** | 305296 | 465 | 0.21 | 1.37 (1.21-1.54) | 1.24 (1.09-1.40) | 1.22 (1.07-1.38) |
| **D5 (<10.16)** | 223623 | 351 | 0.22 | 1.41 (1.23-1.61) | 1.25 (1.09-1.43) | 1.21 (1.06-1.39) |
| **D6 (<15.56)** | 156445 | 277 | 0.24 | 1.59 (1.38-1.84) | 1.38 (1.20-1.60) | 1.33 (1.15-1.54) |
| **D7 (<24.16)** | 109786 | 189 | 0.24 | 1.55 (1.32-1.83) | 1.33 (1.13-1.58) | 1.25 (1.06-1.48) |
| **D8 (<37.82)** | 77594 | 167 | 0.30 | 1.95 (1.64-2.32) | 1.67 (1.40-1.98) | 1.51 (1.27-1.80) |
| **D9 (<59.51)** | 55255 | 119 | 0.30 | 1.96 (1.61-2.39) | 1.68 (1.38-2.05) | 1.44 (1.17-1.76) |
| **D10 (**≥**59.51)** | 41295 | 127 | 0.43 | 2.84 (2.35-3.45) | 2.49 (2.05-3.02) | 1.85 (1.50-2.27) |
| ***P* - value** | | | | <0.001 | <0.001 | <0.001 |

Multivariable adjusted model included age, sex, hypertension, diabetes mellitus, dyslipidemia, heart failure, prior ischemic stroke, prior myocardial infarction, chronic obstructive pulmonary disease, chronic kidney disease, sleep apnea, hyperthyroidism, and low income.

Model 1: unadjusted

Model 2: age and sex

Model 3: age, sex, smoking, alcohol consumption, regular exercise, diabetes mellitus, Hypertension, dyslipidemia, heart failure, prior ischemic stroke, prior myocardial infarction, chronic obstructive pulmonary disease, chronic kidney disease, sleep apnea, hyperthyroidism, and low income.

Abbreviations: CI, confidence interval; FLI, fatty liver index; HR, hazard ratio; IR, incidence rate; PY, person-years.
